# Blood Glutamate Levels in Autism Spectrum Disorder: A Systematic Review and Meta-Analysis

**DOI:** 10.1371/journal.pone.0158688

**Published:** 2016-07-08

**Authors:** Zhen Zheng, Tingting Zhu, Yi Qu, Dezhi Mu

**Affiliations:** 1 Department of Pediatrics, West China Second University Hospital, Sichuan University, Chengdu 610041, China; 2 Key Laboratory of Obstetric & Gynecologic and Pediatric Diseases and Birth Defects of Ministry of Education, Sichuan University, Chengdu, 610041, China; 3 Department of Pediatrics and Neurology, University of California San Francisco, San Francisco, CA 94143, United States of America; Chiba University Center for Forensic Mental Health, JAPAN

## Abstract

**Objective:**

Glutamate plays an important role in brain development, neuronal migration, differentiation, survival and synaptogenesis. Recent studies have explored the relationship between blood glutamate levels and autism spectrum disorder (ASD). However, the findings are inconsistent. We undertook the first systematic review with a meta-analysis of studies examining blood glutamate levels in ASD compared with controls.

**Methods:**

A literature search was conducted using PubMed, Embase, and the Cochrane Library for studies published before March 2016. A random-effects model was used to calculate the pooled standardized mean difference (SMD) of the outcomes. Subgroup analyses were used to explore the potential sources of heterogeneity, and the publication bias was estimated using Egger’s tests.

**Results:**

Twelve studies involving 880 participants and 446 incident cases were included in this meta-analysis. The meta-analysis provided evidence for higher blood glutamate levels in ASD [SMD = 0.99, 95% confidence interval (95% CI) = 0.58–1.40; *P* < 0.001] with high heterogeneity (I^2^ = 86%, *P* < 0.001) across studies. The subgroup analyses revealed higher glutamate levels in ASD compared with controls in plasma [SMD = 1.04, 95% CI = 0.58–1.50; *P* < 0.001] but not true in serum [SMD = 0.79, 95% CI = -0.41–1.99; *P* = 0.20]. Studies employing high performance liquid chromatography (HPLC) or liquid chromatography-tandem mass spectrometry (LC-MS) assays also revealed higher blood glutamate levels in ASD. A sensitivity analysis found that the results were stable, and there was no evidence of publication bias.

**Conclusions:**

Blood glutamate levels might be a potential biomarker of ASD.

## Introduction

Autism spectrum disorder (ASD) is a neurodevelopmental disorder characterized by abnormalities in social interactions, impairments in language and communication, restrictive or repetitive interests, and stereotyped behaviors and movements [1]. ASD includes autistic disorder, Asperger syndrome, and pervasive developmental disorder not otherwise specified [1]. The prevalence of ASD is 11.3 per 1,000 with a male-to-female ratio of 3–4:1 over the last decade [2]. There is growing evidence that ASD may be influenced by genetic, neurological, environmental and immunological factors [3, 4]. However, the underlying mechanism of ASD has not yet been identified. Receiving diagnosis at an early stage of development could contribute to the acquisition of optimized coping strategies for ASD. Diagnosis of ASD is based solely on complex behavioral abnormalities, which are not evident until approximately 12–18 months of age [5, 6]. The abnormal behaviors are often overlooked in early life, even by professionals who are employed in pediatric healthcare. Furthermore, individuals with ASD vary enormously in their clinical manifestations, severity and response to treatment. This complexity is urging an intensive search to identify biomarkers to aid clinicians in achieving an early and more precise diagnosis [7].

Glutamate, the major excitatory neurotransmitter, is ubiquitous throughout the central nervous system. It plays an important role in brain development, affecting neuronal migration, differentiation, survival and synaptogenesis [8]. Glutamate is also involved in general cognitive functions such as memory and learning [9]. However, excess glutamate has been shown to be a potent neurotoxin that leads to neuronal cell death and plays a role in the pathophysiology of some neuropsychiatric disorders [10]. Recently, evidence have implicated that glutamatergic neurotransmission plays an important role in the pathophysiology of ASD [11, 12].

Although glutamate does not readily cross the blood-brain barrier [13], it is reported that glutamate levels in the blood and central nervous system are closely correlated [14]. Thus, the glutamate levels in the blood could be used to reflect the glutamate levels in the brain. This indirect measure of glutamate activity is much easier to perform than directly measuring glutamate in the central nervous system, which offers the possibility that glutamate concentrations in the blood are useful as a possible biological marker for ASD.

The “periphery as a window to the brain” concept has led to an ever-increasing number of clinical studies assessing blood glutamate levels in ASD. However, the results regarding blood glutamate levels in ASD are inconsistent. Some studies have reported that plasma glutamate levels is significantly higher in ASD compared with healthy controls [14, 15, 16], whereas other studies have indicated no significant difference in glutamate levels between individuals with ASD and controls [17, 18].

Thus, we conducted a systematic review of studies assessing blood glutamate levels in ASD and controls, followed by a series of meta-analyses, to provide an overall estimate of the effect size and between-study heterogeneity for the association between blood glutamate levels and ASD.

## Materials and Methods

### Literature search

Two authors searched PubMed, Embase and the Cochrane Library for relevant articles published before March 2016 using Medical Subject Heading (MeSH) terms and the following free text terms: “ASD”, “autism”, “autism spectrum disorder”, “autistic disorder”, “Asperger syndrome”, “pervasive developmental disorder”, and “glutamate”, “glutamic acid”, “aminoglutaric acid”, combined with “peripheral”, “levels”, “serum”, “plasma”, “blood”, “platelets”, and “red blood cells”. In addition, the references of the included articles and previous meta-analyses were searched manually to identify additional studies.

We restricted the search to human studies published in English. The titles and abstracts of the retrieved studies were reviewed to exclude studies that were clearly irrelevant. Then, two authors independently read the full text of the remaining studies to assess their eligibility according to the inclusion criteria. Disagreements about the inclusion/exclusion of a study were resolved by a third author, who independently examined the studies, and a consensus was reached.

### Study selection

Studies were eligible for the analysis if they met all the following criteria: (1) they investigated the association between blood glutamate levels and ASD in vivo, and (2) they provided the mean and standard deviation (SD) for raw data or they provided the median and interquartile range (IQR).

The exclusion criteria for the study were as follows: (1) reviews, case reports, case-only studies, animal studies, simple commentaries; (2) overlapping publications; (3) publications lacking measures of blood glutamate levels, including pharmacological, genetic, brain imaging, and post-mortem studies; (4) ASD with other comorbid conditions; and (5) studies showing glutamate levels in dot plot and histogram format but not providing numerical results after contacting the authors.

### Data extraction

Two authors extracted data from the included articles, which included the following: the first author’s name, publication year, country of region, number of cases and controls, age (mean ± SD), the percentage of females and males, analytical technology, biomaterial, glutamate (mean ± SD), unit of measure and adjusted confounders. Glutamate levels were measured from plasma and serum, and different units of measurement were used across studies, thus limiting their comparability. Therefore, we report all glutamate levels in μmol/l. If the data were presented in the format median (IQR), then the formula “IQR/ 1.35” was used to calculate the SD [19]. If participants overlapped between studies, the one with largest sample size was included in the meta-analysis.

### Quality evaluation

Two authors independently assessed the quality of each included study using the Newcastle-Ottawa Quality Assessment Scale (NOS), and with a maximum of 9 points, to determine the quality of selection, comparability, exposure, and outcomes of the study participants. We divided the study quality into three categories: (1) high quality (scored 7–9); (2) moderate quality (scored 4–6); and (3) low quality (scored 0–3). Disagreements were resolved through mutual discussion.

### Statistical analysis

The standard mean difference (SMD) was used to assess the association between blood glutamate levels and ASD. We pooled the SMD across studies using the Mantel-Haenszel formula (fixed-effect model) or the DerSimonian-Laird formula (random-effect model). A fixed-effect model was chosen when low heterogeneity existed; otherwise, a random-effect model was adopted. Heterogeneity across the studies was tested using the I^2^ and Q statistic, which is a quantitative measure of inconsistency across studies, with suggested thresholds for low (25%—50%), moderate (50%—75%) and high (> 75%) heterogeneity. The Q statistic was considered significant if *P* < 0.1, and I^2^ > 50% indicated high heterogeneity. The results of the analyses are shown in the forest plots.

A potential publication bias was assessed by visual inspection of the funnel plot. Egger’s tests were used to estimate the severity of publication bias, with *P* < 0.05 considered statistically significant.

We conducted subgroup analyses in studies to examine the sources of potential heterogeneity based on biomaterial (plasma or serum), analytical technology (high performance liquid chromatography [HPLC] or liquid chromatography-tandem mass spectrometry [LC-MS]), and geographic location (Asia or other locations).

The unrestricted maximum likelihood random-effects meta-regressions of the effect size were performed with mean age, gender (%male), sample size, biomaterial and publication year as moderators to determine whether these covariates influenced the effect size.

We carried out the sensitivity analysis by removing studies one by one and comparing the SMD of the remaining studies to the SMD for all studies. Statistical analyses were performed using Stata 12.0 (Stata Corp, College Station, Texas, USA) and Cochrane Collaboration Review Manager 5.1.2 (Cochrane Collaboration, Oxford, UK) software.

## Results

### Literature search

**Fig 1:**
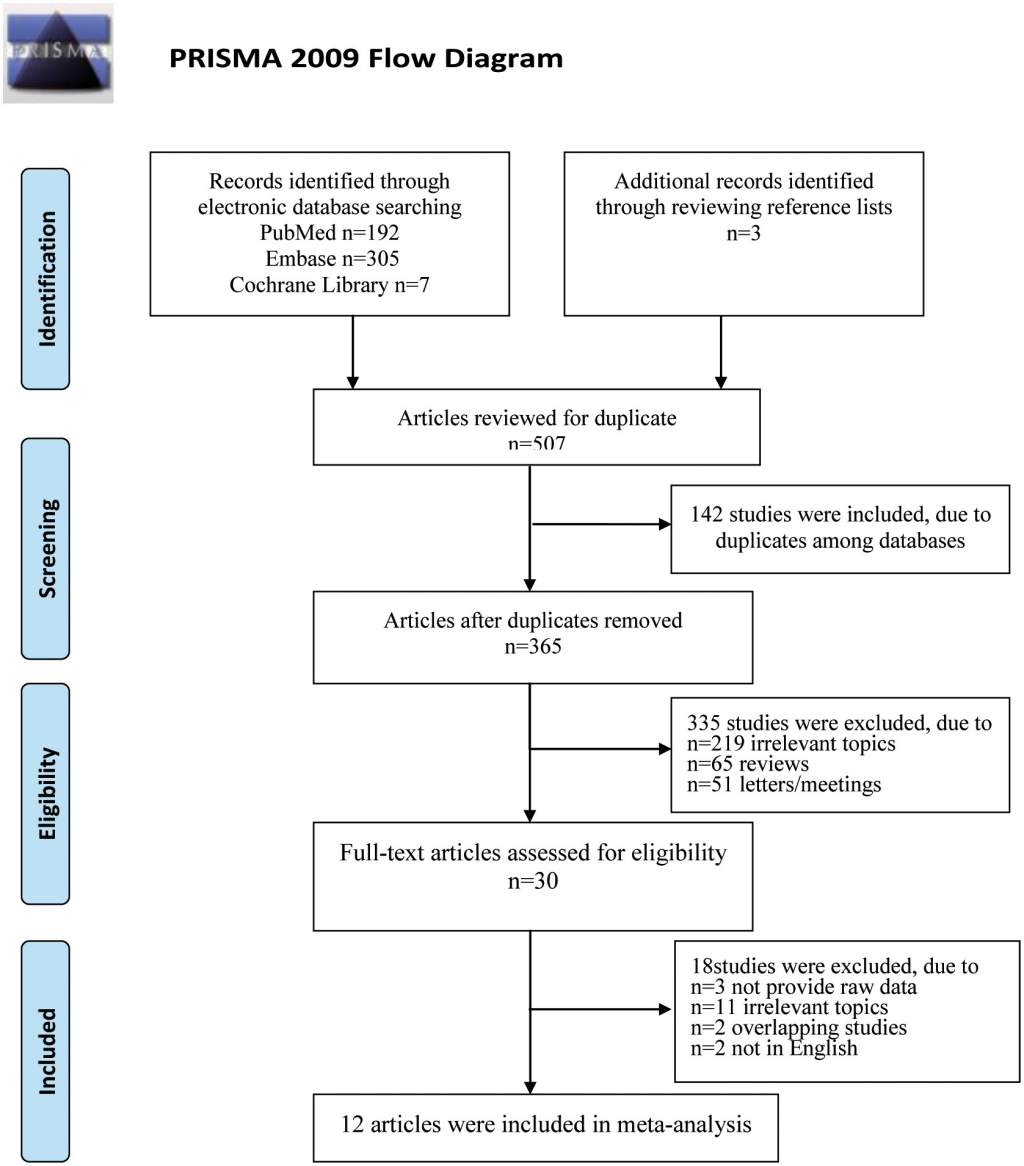
Flow chart of the study selection process for eligible studies in the systematic review. *From*: Moher D, Liberati A, Tetzlaff J, Altman DG, The PRISMA Group (2009). *Preferred Reporting I*tems for *S*ystematic Reviews and *M*eta-*A*nalyses: The PRISMA Statement. PLoS Med 6(6): e1000097. doi: 10.1371/journal.pmedl000097 For more information, visit www.prisma-statement.org.

A total of 507 citations were identified in the initial search, with 192 from PubMed, 305 from Embase, seven from the Cochrane Library and three from reviewing references. After excluding 142 duplicate studies, 219 with irrelevant topics, 65 reviews and 51 letters/meetings, 30 papers on blood glutamate levels in ASD were identified and subjected to a detailed evaluation. Subsequently, three reports without raw data after contacting the authors were excluded, and eleven studies were excluded because of irrelevant outcomes. Two reports were excluded as overlapping studies and another two reports were excluded because they were not published in English. Finally, twelve studies fulfilled all the inclusion criteria, which altogether included 880 participants and 446 incident cases in this meta-analysis. A detailed flow chart of the search and selection process is presented in Fig 1.

### Study characteristics

The characteristics of the twelve selected studies are presented in Table 1. All the studies were published between 1995 and 2016. Twelve studies involving 880 participants and 446 incident cases were included in this meta-analysis. Three studies were conducted in the United States [17, 20, 21], two in China [15, 22], two in Japan [23, 24], two in Egypt [14, 18], one in Saudi Arabia [25], one in India [16], and one in Italy [26]. The sample sizes varied widely, ranging from 10 [14] to 138 [16] ASD individuals and from 10 [14] to 138 [16] controls. Similarly, the mean age of ASD and control individuals varied broadly, ranging from 3.69±1.24 [15] to 22.2±2.2 [24] years. The systematic review identified two different biomaterials used for glutamate assays: plasma [14–18, 20–23, 25] and serum [24, 26]. Moreover, seven studies performed glutamate assessment using HPLC as the analytical procedure [16, 18, 20, 23–26], whereas four adopted LC-MS [14–15, 21–22].

**Table 1:** Characteristics of the twelve studies included in the meta-analysis.

Author Year	Country	Sample size ASD controls	Age(year) Mean±SD(range) ASD controls	Sex(F/M) ASD controls	Analytical technology	Biomaterial	Glutamate Mean±SD ASD controls	Unit of measure	Adjusted confounders
Adams, 2011	America	55; 44	10±3.1; 11±3.1	6/49; 5/39	HPLC	Plasma	0.65±0.15; 0.55±0.13	μmol/l	Age, gender, geographical distribution
Arnold, 2003	America	24; 24	Nr	Nr	Nr	Plasma	51±32; 48±15	μmol/l	Age, gender
Cai, 2016	China	51; 51	3.69±1.24; 3.69±1.24	9/42; 9/42	LC-MS	Plasma	36.1±8.3; 23.6±4.2	μmol/l	Age, gender
ElBaz, 2014	Egypt	20; 20	4.65±1.67; 4.65±1.67	1/19; 11/9	HPLC	Plasma	62.65±88.57; 56.13±62.35	μmol/l	Age, gender
D’Eufemia, 1995	Italy	40; 46	7–17; 5–15	13/17; 19/27	HPLC	Serum	77300±24500; 72400±21200	μmol/l	Nr
Hassan, 2013	Egypt	10; 10	11.4±2.7; 11.3±2.7	4/6; 5/5	LC-MS	Plasma	37±9.17; 20.3±3.65	μmol/l	Age, gender
Naushad, 2013	India	138; 138	4.4±1.7; 4.4±1.6	18/120; 18/120	HPLC	Plasma	120±89; 83±35	μmol/l	Age, gender, ethnicity, geographical distribution
Shimmura, 2011	Japan	23; 22	13.5±2.5; 12.2±2.4	0/23; 0/22	HPLC	Plasma	27.9±7.4; 20.9±4.5	μmol/l	Age, IQ, BMI
Shinohe, 2006	Japan	18; 19	21.2±2.1; 22.2±2.2	0/18; 0/19	HPLC	Serum	89.2±21.5; 61.1±16.5	μmol/l	Age, gender
Shmais, 2012	Saudi Arabia	20; 20	8±4; 7.5±3.5	2/18; 4/16	HPLC	Plasma	152.8±16.77; 111.9±4.63	μmol/l	Age, gender
Tirouvanziam, 2012	America	27; 20	7±2.3; 7.3±2.5	6/21; 11/9	LC-MS	Plasma	97.2±41.8; 74.8±35.8	μmol/l	Age, ethnicity
Tu, 2012	China	20; 20	3.46±0.56; 2–6	3/17; Nr	LC-MS	Plasma	45.6±9.1; 38.9±7.5	μmol/l	Age, gender

Abbreviation: F/M = female/male; HPLC = high performance liquid chromatography; LC-MS = liquid chromatography-tandem mass spectrometry; Nr = no reported.

### Meta-analysis for peripheral glutamate levels in ASD

**Fig 2:**
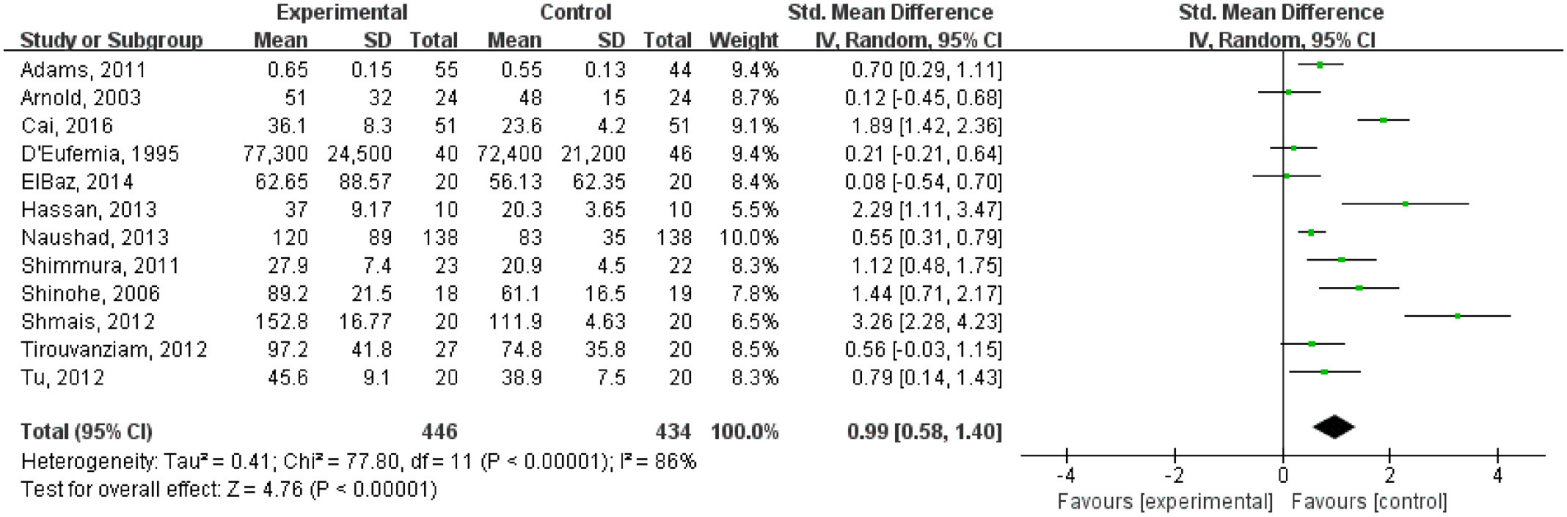
Forest plot for the random-effects between-group meta-analysis of blood glutamate levels in persons with ASD and controls.

By pooling the estimates from twelve studies, a significantly higher level of glutamate was found in ASD compared with controls [SMD = 0.99, 95% CI = 0.58–1.40; *P* < 0.001]. However, there was significant statistical heterogeneity across studies (I^2^ = 86%, *P* < 0.001) (Fig 2).

### Quality evaluation

The results of the quality assessment of the included studies are shown in Table 2. Twelve studies are of high quality, with an average score of 7.33.

**Table 2:** Quality assessment of the included studies by the Newcastle–Ottawa Scale.

Publication year	Study design	Selection	Comparability	Exposure/Outcome	Total scores
Adams, 2011	Cross-section	★★★	★★	★★	7
Arnold, 2003	Cross-section	★★★	★★	★★	7
Cai, 2016	Cross-section	★★★★	★★	★★	8
ElBaz, 2014	Case-control	★★★	★★	★★	7
D’Eufemia, 1995	Cross-section	★★★	★★	★★	7
Hassan, 2013	Case-control	★★★	★★	★★	7
Naushad, 2013	Cross-section	★★★	★★	★★	7
Shimmura, 2011	Cross-section	★★★★	★★	★★	8
Shinohe, 2006	Cross-section	★★★★	★★	★★	8
Shmais, 2012	Cross-section	★★★	★★	★★	7
Tirouvanziam, 2012	Cross-section	★★★★	★★	★★	8
Tu, 2012	Cohort	★★★	★★	★★	7

### Publication bias

**Fig 3:**
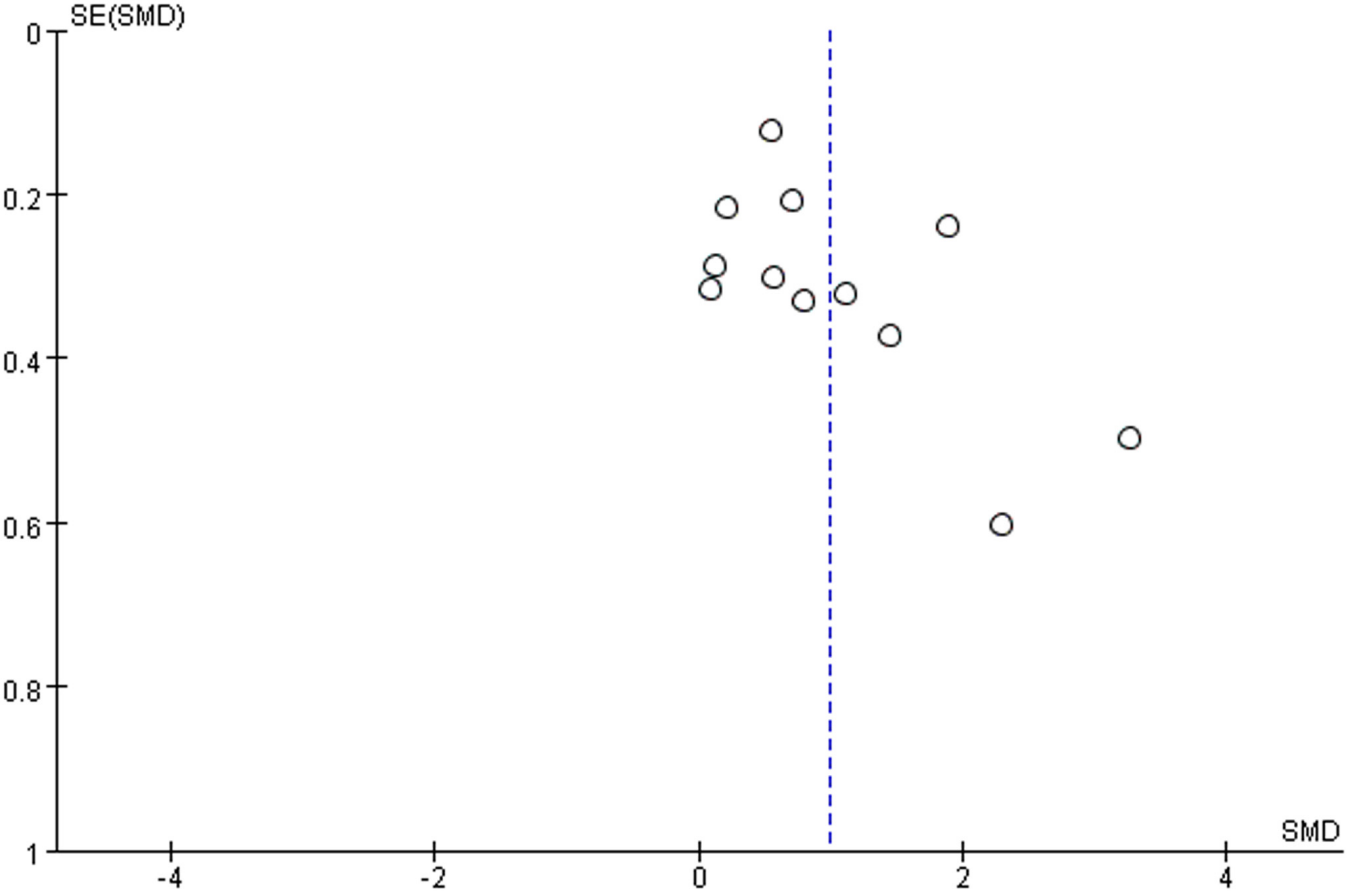
Funnel plot for the random-effects between-group meta-analysis on blood glutamate levels in persons with ASD and controls.

Visual inspection of the funnel plot indicated some asymmetry for the included studies (Fig 3). However, Egger’s tests did not show significant evidence of a publication bias among the included studies (*P* = 0.107).

### Subgroup analysis

Of the twelve studies included in this meta-analysis, ten included studies describing glutamate levels assessed in plasma. Two studies reported results assessed in serum. The pooled SMD was 1.04 (95% CI = 0.58–1.50, *P* < 0.001) for glutamate levels assessed in plasma and 0.79 (95% CI = -0.41–1.99, *P* = 0.20) for glutamate levels assessed in serum (Table 3).

**Table 3:** Summary of the results of blood glutamate levels in persons with ASD and controls.

Variables	No. of comparisions	No. of subjects		Meta-regression				Heterogeneity	
		ASD	Controls	SMD	95%CI		P-value	I^2^	P-value
**Biomaterial**									
Plasma	10	388	369	1.04	0.58	1.5	<0.001	87	<0.001
Serum	2	58	65	0.79	-0.41	1.99	0.2	88	0.005
**Analytical technology**									
HPLC	7	314	309	0.93	0.44	1.43	<0.001	86	<0.001
LC-MS	4	108	101	1.32	0.53	2.11	0.001	83	<0.001
**Geographic locations**									
Asia	6	270	270	1.44	0.75	2.13	<0.001	90	<0.001
Others	6	176	164	0.5	0.1	0.9	0.01	66	0.01

Abbreviation: HPLC = high performance liquid chromatography; LC-MS = liquid chromatography-tandem mass spectrometry; No. = number; CI = confidence interval.

We also conducted a meta-analysis for the analytical technology used, either HPLC or LC-MS. The effect size of the differences in the glutamate levels measured in ASD and controls when applying these different analytical technologies was 0.93 (95% CI = 0.44–1.43, *P* < 0.001) for HPLC and 1.32 (95% CI = 0.53–2.11, *P* = 0.001) for LC-MS (Table 3).

Of the twelve studies included in this meta-analysis, six studies were performed in an Asian population, whereas six studies were performed in non-Asian groups. The pooled SMD was 1.44 (95% CI = 0.75–2.13, *P* < 0.001) for glutamate levels assessed in Asian groups and 0.50 (95% CI = 0.10–0.90, *P* = 0.01) for glutamate levels assessed in non-Asian groups (Table 3).

### Meta-regression analysis

We performed meta-regression analyses in an exploratory attempt to identify the sources of heterogeneity among studies and the effect of moderators. There was no relationship between the mean age, gender, sample size, biomaterial or publication year with respect to glutamate levels (Table 4).

**Table 4:** Meta-regression of blood glutamate levels in persons with ASD and controls.

Moderator	No. of comparisions	No. of subjects		Meta-regression				Proportion of variance explained R^2^ analog
		ASD	Controls	Slope	95%CI		P-value	
Age(mean, years)	9	362	344	0.028	-0.125	0.18	0.678	0
Gender(% male)	10	402	390	1.247	-3.255	5.748	0.541	0
Sample size	12	446	434	-0.003	-0.012	0.006	0.497	0
Biomaterial	12	446	434	-0.275	-1.943	1.393	0.721	0
Publication year	12	446	434	0.059	-0.042	0.16	0.225	7.48

Abbreviation: No. = number; CI = confidence interval.

### Sensitivity analysis

The influence of each study on the overall estimate was assessed by removing studies one by one and comparing the pooled estimate from the remaining 11 studies with the pooled estimate from all 12 studies. The results revealed a higher level of peripheral glutamate levels in ASD compared with controls in all 12 analyses, indicating that the removal of any study would not alter the overall results.

## Discussion

Pooling the twelve studies yield 880 participants and 434 incident cases that were included in this meta-analysis. Since there was a significant between-study heterogeneity, a random-effect model was used to compute the pooled estimates. The pooled SMD indicated higher blood glutamate levels in ASD compared with the controls. A sensitivity analysis found that the pooled results were robust. Egger’s tests did not show significant evidence of a publication bias.

Glutamate, the major excitatory neurotransmitter, is ubiquitous throughout the central nervous system. Glutamate is reported to not easily cross the blood-brain barrier [13]. However, studies have shown that glutamate levels in the blood are positively correlated with those in the cerebrospinal fluid of humans [27]. Recently, Hassan et al. [14] reported a highly significant positive correlation between blood glutamate levels and brain glutamate levels. Thus, the blood glutamate levels can be used to reflect the glutamate levels in the brain.

Accumulating evidence suggests that abnormalities in glutamatergic neurotransmission may play a role in the pathophysiology of ASD [11, 12]. In this study, the meta-analysis indicated higher blood glutamate levels in ASD than in controls, which may reveal higher glutamate levels in the brain in ASD when compared with controls. Importantly, higher glutamate levels in the brain have been demonstrated by using proton magnetic resonance spectroscopy [14, 28], which further indicated that glutamate levels in the blood may be a possible biological marker for ASD.

Although the mechanism of increased glutamate in ASD is not entirely known, several mechanisms have been considered. First, elevated glutamate levels in the brain are consistent with evidence suggesting the relationship between a dysregulation of glutamine/glutamate metabolism and increased gliosis in the brains of ASD [23]. The process of gliosis activates astrocytes and/or microglia, which may disturb the regulation of certain types of enzymes and thereby alter the metabolism of glutamate/glutamine [29]. Second, Yip et al. [30] have shown that the levels of glutamic acid decarboxylase, which are involved in converting glutamate to GABA, are reduced in the brains of ASD, resulting in increased levels of glutamate in the brain. Third, glutamate plays an important role in the initiation and spread of seizure activity. Increased glutamate levels may be implicated in the high rates of seizure disorder in ASD [31]. Clinical reports demonstrated that the mood stabilizer valproic acid, which exerts neuroprotective effects against glutamate-induced excitotoxicity, is effective in ASD with seizures [32]. This might indirectly indicate that increased glutamate levels may be involved in the pathogenesis of ASD. Finally, glutamate is important for maintaining functions such as memory, learning, behavior, motor activity, etc. Abnormal glutamate levels might be associated with a wide variety of neurobiological and behavioral alterations in ASD [33].

The subgroup analyses revealed that glutamate levels were significantly higher in individuals with ASD compared with controls when measured in plasma but not in serum. The subgroup of serum was smaller than the group of plasma studies, and the lack of statistically significant differences could therefore represent a type II error or because of other study characteristics shared by studies of serum glutamate. In the subgroup based on geographic locations, a more positive SMD was found among Asian populations. There was a large difference between the SMDs (1.44[0.75, 2.13] vs. 0.5 [0.1, 0.9]) of Asian populations and non-Asian populations. This difference may be induced by environmental factors, genetic factors, lifestyle and economic status.

Our findings indicate some advantages. First, this is the first comprehensive meta-analysis conducted to assess the association between blood glutamate levels and ASD. Second, the sensitivity analysis did not alter the final results, which increased the robustness of our findings. Third, there was no significant publication bias detected, suggesting that our results are reliable.

However, there are some limitations to our meta-analysis. First, the sample size of participants in the included studies was small. More comprehensive studies with large samples that may clarify the role of higher blood glutamate levels in the disease process are warranted. Second, dietary intake and absorption may influence blood glutamate levels. Particular eating habits, dietary patterns [34] and functional gastrointestinal abnormalities [35] have been observed in ASD. These factors may contribute to the altered blood glutamate levels in ASD. Some included studies did not account for overall diets or for the presence of any gastrointestinal symptoms, which future studies will need to address. Third, detailed information on medication was not reported in some studies, and medication may also influence blood glutamate levels in ASD. Thus, future research should take into consideration the possible effect of medication on blood glutamate levels. Fourth, we did not assess the correlation between the severity of ASD and glutamate levels because only a few studies performed analyses on the clinical severity of ASD and blood glutamate levels. Future studies on the association between these factors are needed. Finally, there was significant heterogeneity in the analyses. Although we performed random-effects, subgroup analyses and regression analyses, these parameters could not explain the heterogeneity. Residual confounding factors across studies, including dietary habits, medical effects, length of illness, remain a cause for concern in this meta-analysis. Heterogeneity was still an inevitable problem that may affect the precision of the overall results.

In conclusion, this meta-analysis suggests that blood glutamate levels are higher in ASD compared with controls and further suggests that blood glutamate levels may serve as a potential biomarker in the diagnosis of ASD. More comprehensive studies with large samples are needed to provide more conclusive results. The effects of medications on peripheral glutamate levels and the correlation between the clinical symptoms in ASD and peripheral glutamate levels need further investigation.

## Supporting Information

S1 File PRISMA checklist. (DOC)
